# The influence of psychosocial factors on productivity when implementing new office designs - a longitudinal explorative study in the Swedish public sector

**DOI:** 10.1186/s12889-025-22953-4

**Published:** 2025-05-23

**Authors:** Åsa Stöllman, Magnus Svartengren, Erik Lampa, Teresia Nyman

**Affiliations:** 1 Department of Medical Sciences, Occupational and Environmental Medicine, Uppsala University, Uppsala, SE- 751 85 Sweden; 2 Department of Occupational and Environmental Medicine, Uppsala University Hospital, Uppsala, SE-751 85 Sweden; 3 Department of Medical Sciences, Clinical Epidemiology, Uppsala University, Uppsala, SE-751 85 Sweden

**Keywords:** Psychosocial factors, Work environment, Office changes, Productivity, Leadership, Relational justice

## Abstract

**Background:**

In implementation of new office designs the process is of great importance for a successful outcome in terms of a healthy work environment and productivity. Knowledge regarding psychosocial factors needs to be applied early in the implementation process. The study’s objective was to explore potential associations between pre-existing psychosocial factors before implementation of open plan office solutions, and self-reported production loss due to work environment problems after the implementation.

**Method:**

Two departments in two large public organisations were included in the study; organisation A with 598 employees across twelve units, and organisation B with 304 employees across six units. At baseline and follow-up, the participants completed a questionnaire. Paired analyses regarding office types, psychosocial factors and production were performed. Ordinal logistic regression was used for analysing associations between baseline psychosocial factors; communication, leadership, relational justice, and self-reported production loss due to work environment problems at follow up.

**Results:**

Several pre-existing psychosocial factors before implementation of new office designs were found to be associated with self-reported production loss due to work environment problems at follow-up. Collaboration within units emerged as a significant factor in both organisations, where a more favourable collaboration was seen as a protective factor, suggesting its importance. The results were more pronounced for Organisation B, where control of decisions, collaboration between units, and all three factors related to leadership: support from superiors, relational justice, and trust in management were significant. The direction of the associations for these variables were the same in Organisation A, but the results did not reach statistical significance. The reverse was seen for quantitative demands restoration from sleep, and attitudes towards relocation where statistically significant associations were found solely in Organisation A.

**Conclusions:**

Although several psychosocial factors seemed to matter for a positive result of an office change, the present study contributes primarily with the knowledge that change always takes place in a unique context for each specific organisation. The mechanisms are interconnected and complex, concerning for instance organisational culture and structure, characteristics of work tasks, and differences in the implementation process.

**Supplementary Information:**

The online version contains supplementary material available at 10.1186/s12889-025-22953-4.

## Introduction

Worldwide in the last decades, a growing trend in work place design is the shift from individual office solutions (i.e. cell offices), into different kinds of open plan solutions, such as traditional open plan offices (employees has their own desk in an open plan office with more than 3 seats) or more modern solutions such as flex offices (open plan office and not their own desk) or activity-based office design (optional desks and workplaces in open plan design, with various functions and interactions to choose between, suitable for different work tasks).

The trend started in the private sector, but such changes of office design have also been implemented in organisations within the public sector, i.e. in healthcare and in governmental organisations such as regional and municipality administrations [1, 2].

The transformation to open plan office solutions have mainly been driven by economic incitements (to reduce cost for facility), but with the development of the activity-based office concept also as an adaption to new conditions in working life, referring to the concept of “new ways of working” to facilitate communication and creativity [3, 4]. Besides the intended economic advantages of implementing activity-based offices, the office industry claims that activity-based office solutions also benefits work performance and collaboration. However, previous research can only partly confirm such statements [5–7].

Distraction and satisfaction are two well-studied factors in office research [8] as distraction is a frequently reported problem in open plan office environments, especially regarding concentration intensive tasks. In activity-based offices, the solution to this problem is to design the layout of the office with specific quiet zones. Satisfaction with activity-based offices seems to be positively related to switching work zone frequently [9]. However, studies have shown that employees tend not to change work zones to the extent that they benefit from the activity-based offices design advantages. Instead, social needs tend to be prioritised rather than planning how to use the different work zones during the workday [10].

Hence, the implementation of a new office design may also be compared to an organisational change itself, not in terms of work content, but the work tasks might need to be carried out in a different way. Leadership in open plan offices also requires a different approach, a Swedish study (Larsson, 2022) examined employees’ experiences of leadership behaviours when the office design was changed from cell or open plan offices to activity-based offices [11]. The employees that changed from open plan offices to activity-based offices rated relation-, structure- and change-oriented leadership behaviours significantly lower one year after the change. A participative approach in the implementation process, by engaged leadership, and by commitment in the planning of the change [12] has shown to be of great importance for a successful outcome [6, 13]. Organisational change competence should be included in the planning of the new office, early on in the implementation process. Conditions that contribute in developing organisational change competence are management and change agents; employees attitudes towards change, information and communication and participation and opportunities for learning in change processes [14].

Besides the impact of the implementation process, it is well known that psychosocial factors; leadership and perceived fairness in the organisation, job resources such as support, satisfactory communication and collaboration (both within a team and throughout the organisation), a balance between job demand and job control, clarity of work tasks and work role, plays important roles when it comes to employee health [15–20]. The relocation to an open plan office may have various effects on the psychosocial work environment, influencing quantitative demands, control of decisions, support from superiors and from co-workers, and role clarity. The proposed mechanisms and their impact may vary, but some general considerations are for example that with an increase in distractions and interruptions, it might potentially lead to an increase in workload due to difficulties in focusing. It may also lead to a perceived lack of privacy, potentially diminishing employees’ sense of control over their workspace and decisions, and also it may hinder social interactions which might limit meaningful communication and support. With this said, well-functioning, stable and proactive pre-existing psychosocial factors may likely be of great importance for the implementation process [21].Employee health affects work-related productivity loss [22], and a factor that may influence both health and productivity is sleep disturbances, which in previous studies has been shown an early predictor of health problems. Sleep disturbances are a primary health concern for workers in the western world. Adverse psychosocial work factors are related to poor sleep, and hence may have an impact on sleep disturbances [23].

Within organisational research, the Demand Control Support Model [15, 16] is a well-established theory that explains how employee health is positively related to job control and to social support in the workplace and negatively affected by high work demands [24]. This framework has been further developed into the expanded Job Demand-Resources (JD-R) model, which not only explains how job demands contribute to stress but also recognises that various job resources can both mitigate negative effects and enhance work engagement [19, 20]. Another theory within this domain is the organisational justice model. This model describes four dimensions: distributive justice, procedural justice, interpersonal (or relational) justice and informative justice [25]. The present study focuses on the relational justice dimension, which emphasises the relationship between the manager and the employees; i.e. how the manager handles the employees’ personal views and rights; and if the employees are treated impartially, truthfully and with kindness. Relational justice has been shown to be linked to several health outcomes [26–28] and studies show that how fairly employees are treated can predict future ill health such as diminished self-rated health and burnout [29–31]. Also, it has been shown that employees in companies with a higher relational justice index had higher ratings of both health and well-being [18, 27].

Studies have shown an association between the psychosocial work environment and performance outcomes such as productivity. The performance of the organisation depends on the performance of the employees; therefore, assessing performance and production loss are valuable. A method to subjectively measure performance on an organisational level, is to use questionnaires of self-reported productivity such as production loss due to ill-health or work environment deficiencies [32]. Earlier research shows that employees that report psychosocial work environment problems (inequality and high decision demands) also report high levels of production loss, compared to individuals who experience a satisfactory psychosocial work environment [33]. Lohela-Karlsson, et al. [34] also suggests that perceived work environment-related production loss may be used to evaluate the effects of organisational changes. Furthermore, factors linked to the work environment seem to have a greater impact on production loss than factors linked to health [33].

To be able to implement open plan office solutions that will benefit both employee health and productivity over time, knowledge regarding what factors that contribute to healthy work places in general, need to be applied already in the implementation process. However, earlier research that have focused on psychosocial factors, health effects or productivity, has mostly been performed when the new office solution already had been implemented. Hence, it is of interest to investigate how the pre-existing psychosocial work environment may influence the effects of a forthcoming change in office design in terms of both psychosocial factors, health and productivity in an organisation.

The hypothesis of this study posits that psychosocial factors in the work environment prior to transition to an open plan office will significantly influence the overall success of the relocation.

Specifically, the study examines whether psychosocial factors can proactively influence employees’ ability to adapt to and experience the new office environment without the new office designs posing a negative influence on the work environment.

To elaborate, the study seeks to understand if employees who have a history of being well-treated are better equipped to adjust to the changes brought about by the open plan office, thereby contributing to a smoother transition. For instance, the study considers whether perceived fairness and support from supervisors and colleagues play a role in shaping attitudes toward the relocation. Additionally, it explores whether a positive perception of fairness and good treatment contributes to resilience and satisfaction with the new work environment.

Furthermore, the study investigates whether supervisors providing clear communication, guidance, and support can help employees feel more confident and less anxious about the relocation. Given that open plan offices can impact communication patterns with increased noise and interruptions, the research considers whether a well-established collaboration practice beforehand makes it easier for employees to adapt to transparency and open communication in such a setting.

Finally, the study explores whether clearly defined roles contribute to employees’ understanding of their responsibilities and expectations, thereby preventing confusion, minimizing conflicts, and fostering an efficient work environment.

Hence, the objective of the study was to explore potential associations between pre-existing psychosocial factors before implementation of open plan office solutions, and self-reported production loss due to work environment problems after the implementation.

## Materials and methods

This cohort study has an explorative approach with a longitudinal design and is part of a large follow up-study investigating the implementation of new office solutions in two large organisations within Swedish public sector. The overall aim of the overarching project is to explore how implementations of new office solutions can be successful in creating and sustaining a healthy and sustainable organisation. In the present study the STROBE checklist has been used in the preparation of the manuscript [35].

Further, the present study focuses on exploring how psychosocial factors in organisations prior to an implementation of new office solutions, may affect the subjective production loss after relocation.

### Study setting and participants

Two management/administrative departments in two large Swedish public organisations were included in the study; the management/administrative department in organisation A with 598 office workers across twelve units, and the management/administrative department in organisation B with 304 office workers across six units. The whole organisation A had a total of 6 500 employees and the whole organisation B had a total workforce of 12 000 employees. Hence forward, the two departments will be referred to as “Organisation A” and “Organisation B”. Both departments, situated in a densely populated area in the central part of Sweden experiencing economic growth, were in the process of relocating to newly constructed office buildings equipped with modern office solutions that’s been selected by the organisations. The relocation made it possible to bring the units closer together, from being spread out geographically. Both the old and the new office buildings are centrally located in the same city, hence there was no major change of employees’ commuting patterns.

The implementation process had been dispersed over an extended period in both departments, involving negotiations with the union to determine the most suitable office solution to meet the organisations’ needs, among others the need of increasing flexibility in office use to save space and drop costs. Initially, both organisations had planned a complete transition to activity-based offices, but this plan was modified following negotiations with the union. Throughout the process, both organisations facilitated activities for staff participation, ensuring that they were informed and provided opportunities to share their opinions, wishes, and ideas.

In Organisation A, a project group for the relocation was formed, led by a top manager within the organisation serving as the project leader, and included all unit-level managers. In organisation B, the project group consisted of managers and employees representing the various units. This group was led by an external consultant.

At baseline, Organisation A employed 598 office workers across twelve units, while Organisation B had 304 office workers distributed among six units. These units primarily focused on management, administrative support, human relations services, IT support, and other support functions. For Organisation A, all twelve units were included in the study, while in Organisation B, one unit (*n* = 40) that was initially intended to relocate into the new building did not participate. This was due to the unit’s responsibilities involving confidential telephone service 24/7, which were incompatible with an open plan office. Consequently, this unit was excluded from the study population. The unit sizes ranged from 8 to 125 employees.

At baseline, 418 out of 598 participants in Organisation A responded (70%), and in Organisation B, 191 out of 264 participants responded, resulting in a response rate of 72%. During the follow-up period, Organisation A saw an increase in office workers to 620, while in Organisation B the number of employees decreased to 261. The number of respondents at follow-up was 398 in Organisation A. The corresponding numbers in Organisation B was 169, resulting in a response rate of 64% in both organisations. The questionnaire was sent to all employees at both baseline and follow-up. The respondents are described in Table 1.

For Organisation A, baseline data collection occurred in December 2016, with follow-up data collected in September 2018. The relocation occurred in August 2017. Regarding Organisation B, baseline data collection took place in April/May 2016, and follow-up data was collected in September 2017. The relocation for Organisation B occurred in September 2016. The original plan was to maintain the same time interval between baseline and follow-up measurements in both organisations. However, due to varying processes, different measurement points for baseline were implemented.

**Table 1:** Descriptive information of the study population at baseline and at follow-up

		Organisation A		Organisation B	
		***Baseline***	***Follow-up***	***Baseline***	***Follow-up***
		***N (%)***	***N (%)***	***N (%)***	***N (%)***
Women/men		268/150 (64/36)	269/129 (68/32)	128/63 (67/33)	121/48 (72/28)
Employment > 3 years		279 (68)	276 (69)	118 (62)	104 (62)
Age	*≤* 40 years	99 (24)	80 (20)	47 (25)	41 (24)
	41–50 years	157 (38)	146 (37)	63 (33)	50 (30)
	51–60 years	128 (31)	137 (35)	53 (28)	59 (35)
	> 60 years	31 (7)	32 (8)	27 (14)	18 (11)
Education	High school level	63 (15)	57 (14)	31 (16)	30 (18)
	University level	337 (85)	323 (82)	158 (84)	137 (81)

Number of respondents (*N*), age at baseline (years), employment > 3 years, and education level at baseline

Not all respondents answered all questions

### Data collection

At baseline and at follow-up, the participants completed an online questionnaire comprising in all 93 questions. In the questionnaire, the participants reported on different aspects of their psychosocial work environment as well as individual factors and subjective productivity. The domains in the questionnaires were chosen based on previous research concerning factors of importance for creating and sustaining a healthy workplace, and were investigated by models and questionnaires well known in the research field (For the Cronbach Alpha values of the indices, see Appendix A, Table A1). The questions used in the present study are presented below.

*Psychosocial factors* were investigated using 18 questions from the QPS-Nordic questionnaire [36, 37]. In all, six indexes were constructed exploring the following domains:

1. “Quantitative demands” (4 items: Is your work load irregular so that the work piles up?; Do you have to work overtime?; Is it necessary to work at a rapid pace?; Do you have too much to do? ).
2. “Control of decisions” (4 items; If there are alternative methods for doing your work, can you choose which method to use? Can you influence the amount of work assigned to you? Can you influence decisions concerning the persons you will need to collaborate with?; Can you influence decisions that are important for your work? ).
3. “Support from superior” (3 items; If needed, can you get support and help with your work from your immediate superior? If needed, is your immediate willing to listen to your task-related problems? Are your work achievements appreciated by our immediate superior? ).
4. “Support from co-workers” (2 items: If needed, can you get support and help with your work from your co-workers? If needed, are your co-workers willing to listen to your work-related problems? ).
5. “Role clarity” (3 items: Have clear, planned goals and objectives been defined for you? Do you know what your responsibilities are? Do you know exactly what is expected of you at work? ).
6. “Role conflict” (3 items: Do you have to do things that you feel should be done differently? Are you given assignments without adequate resources to complete them? Do you receive incompatible requests from two or more people? ).

*Collaboration within units and between units* were explored using seven questions developed by Rolfö et al. [38]. The rating scale for each of the items ranges from 1 = Very bad to 5 = very good. Two indexes were constructed:

1. “Collaboration within unit” (4 items: How is the productivity within the team? How does intra-team cooperation work? How well do new ideas spread within the team/unit? How well does work-related communication function between the colleagues in your team/unit? ).
2. “Collaboration between units” (3 items: How does cooperation with other teams/units work? How well do new ideas spread to other teams/units? How do you perceive the spreading of information between units all in all?

*Relational Justice* was investigated using the six items that constitutes the relational justice index. The items are formulated to encapsulate how the employees experience to what degree supervisors handle the employees’ personal views and rights, and whether the employees are treated impartially, truthfully and with kindness by their superior [29, 30]. The rating scale for each of the items ranges from 1 = strongly disagree to 5 = strongly agree. The items are formulated as follows:

1. Your supervisor considers your viewpoints.
2. Your supervisor is able to suppress personal biases.
3. Your supervisor provides you with timely feedback about the decisions and their implications.
4. Your supervisor treats you with kindness and consideration.
5. Your supervisor shows concern for your rights as an employee.
6. Your supervisor takes steps to deal with you in a truthful manner.

*Sleep quality and sleep disturbances* were investigated by the use of two indexes regarding sleep quality and restoration from sleep from the Karolinska sleep questionnaire (KSQ short version) [39–41]. The “sleep quality” index contains 4 items (difficulty falling asleep, waking up with difficulty going back to sleep, waking up too early, and having restless/disturbed sleep) and the “restoration from sleep” index contains 3 items (difficulty waking up, waking up feeling unrested, and waking up fatigued). The rating scale for each of the items ranged from 1 = always (5 times or more per week), 2 = most of the times (3–4 times per week), 3 = often (1–2 times per week), 4 = sometimes (several times per month), 5 = seldom (occasionally), to 6 = never.

*Questions regarding the relocation process* were constructed specially for the present study. The employees were asked to rate their “attitude towards relocation before relocation” and “satisfaction with relocation process after relocation”, the rating scale ranging from 0 = Very negative to 10 = very positive. Further, the employees also rated their trust in management before and after relocation, respectively. The rating scale ranged from 1 = Very low or no trust at all, to 5 = very high trust.

*Work environment-related production loss*, (the effect of work environment-related problems on employees’ performance), was investigated using the single-item yes/no question, “Over the past 7 days, have you experienced work environment-related problems at work?”. If given a positive answer, a follow-up question followed: “During the past 7 days, how much did your work environment-related problems affect your performance while you were working?”. The rating scale for this follow-up question ranges from 0 = The work environment problems had no effect on my work, to 10 = The work environment problems completely prevented me from working [22, 32, 33].

### Statistical analyses

The results are based on the answers from the same individuals in both measurements, paired analyses. In organisation A, 418 individuals responded to the baseline questionnaire, rendering a response rate of 70%. At follow-up, 285 (68%) of the same individuals answered the follow-up questionnaire. The corresponding numbers for organisation B was 191 individuals that completed the baseline questionnaire (72%) and at follow-up 113 (59%) of the same individuals completed the follow-up questionnaire. The drop-out analysis of the follow-up questionnaire showed that the non-responders were over represented among the youngest and the oldest age group in both organisations, and slightly over represented among men (See Appendix A, Table A2). Individuals changing jobs, being on sick leave at the time of the data collection or having retired due to age, can be considered plausible reasons for non-response. However, there are no data available to confirm this. No drop-out analysis could be done of the baseline measurements.

The analyses in the present study are based on the respondents that answered both the baseline and the follow-up questionnaire, and held the same position at work at both occasions (employee/manager) (Table 2).

**Table 2:** Descriptive information of the respondents that answered both the baseline and the follow-up questionnaire, and held the same position at work at both occasions

		Organisation A	Organisation B
		***N (%)***	***N (%)***
Employees/managers		236/30 (89/11)	96/13 (89/11)
Women/men		177/89 (67/33)	75/34 (69/31)
Employment > 3 years		178 (67)	68 (63)
Age	*≤* 40 years	58 (22)	25 (23)
	41–50 years	103 (39)	35 (32)
	51–60 years	91 (34)	38 (35)
	> 60 years	14 (5)	11 (10)
Education	High school level	43 (16)	20 (18)
	University level	210 (84)	89 (82)

Number of employees/manager (*N*) (same position at both baseline and follow-up), number of women/men (*N*), age at baseline (years), employment > 3 years, and education level at baseline. Not all respondents answered all questions

To investigate if the exposure to psychosocial factors at baseline (before relocation) differed depending on designated office type, independent sample t-tests comparing cell office and shared office (2-4p) were performed. To investigate if the exposure to psychosocial factors at follow-up (after relocation) differed depending on designated office type, one-way ANOVA analyses and Kruskal–Wallis H tests were executed. To investigate if the exposure to psychosocial factors differed between baseline and follow-up, paired sample t-tests were executed.

The baseline variables regarding psychosocial factors, communication within units and between units, relational justice, sleep quality and sleep disturbances, and questions regarding the relocation process were screened for associations with work environment-related production loss at follow up using ordinal logistic regression [42]. Variables were entered one by one into the model, assuming linearity, with further adjustments for age, sex and work environment-related production loss at baseline. An interaction between the variables and organisation type was forced into all models. This enables organisation specific estimates to be obtained and the ability to test whether or not these differences way exist.

### Ethical approval

The study was approved by the Regional Ethical Vetting Board in Uppsala (DNr 2016/481) and follows the guidelines according to the Declaration of Helsinki (43). Prior to the start of the study, all employees at the participating departments were informed both orally and in writing and gave their written consent in connection with completing the baseline questionnaire. At completion of data collection, the personal information was replaced with a code in the database to guarantee confidentiality. The data was handled in a safe manner being stored according to standard regulations for storage of data at the Uppsala university. Only the researchers had access to the database.

## Results

The objective of the study was to explore potential associations between pre-existing psychosocial factors before implementation of open plan office solutions, and self-reported production loss due work environment problems after the implementation.

The results are presented in the following order. Firstly, the results comparing psychosocial factors at baseline and follow-up are presented, followed by the corresponding results concerning work environment problems and self-reported production loss. Secondly, the analyses concerning associations between psychosocial factors, work environment problems and self-reported production loss is presented. Additionally, the influence of designated office type on psychosocial factors and work environment problems is presented in the last section of the results.

### Psychosocial factors at baseline and follow-up

When comparing psychosocial factors at baseline and follow-up, no discernible patterns of changed exposures could be identified, and only limited differences were observed (see Table 3). In both organisations, trust in management had increased at follow-up (*p* = 0.030), from 3.52 (SD 0.93) to 3.66 (SD 1.00) in Organisation (A) The corresponding figures in Organisation B was 3.32 (SD 0.98) at baseline, to 3.56 (SD 0.99) at follow up (*p* = 0.009). No other statically significant differences were found in Organisation (B) Statistically significant changes regarding other factors were identified in Organisation A, including lower job demands, increased support from superiors, reduced role conflict, improved collaboration within units, and enhanced sleep quality. On an aggregated organisational level, the relocation to the new office design did influence how the psychosocial work environment was perceived differently in the two organisations. However, in both organisations, attitudes towards relocation were generally positive, with a mean value of 6.60 (SD 2.93) for Organisation A and a mean value of 7.28 (SD 2.56) for Organisation B on a scale of 1–10, where 10 indicates the most positive attitude towards the relocation.

**Table 3:** Psychosocial factors at baseline and at follow-up. Stratified by organisation. In the table, the direction of the question is indicated by an arrow, where ↓ = the lower the value the more positive the work environment is perceived. Correspondingly, ↑ (Higher value) indicates that the higher the value, the more positive the work environment is perceived

	Organisation A			Organisation B		
	Baseline	Follow-up		Baseline	Follow-up	
	Mean (SD)	Mean (SD)	*p*-value	Mean (SD)	Mean (SD)	*p*-value
**Quantitative demands (1–5) **↓	3.25 (0.78)	3.16 (0.80)	0.008*	3.26 (0.79)	3.22 (0.75)	0.481
**Control of decisions (1–5) **↑	3.16 (0.71)	3.20 (0.72)	0.362	3.26 (0.74)	3.36 (0.75)	0.068
**Support from superior (1–5) **↑	3.75 (1.02)	3.90 (0.97)	0.011*	3.86 (0.98)	3.70 (0.98)	0.064
**Support from co-worker (1–5) **↑	4.25 (0.80)	4.22 (0.83)	0.564	4.24 (0.78)	4.15 (0.80)	0.296
**Role clarity (1–5) **↑	3.85 (0.87)	3.89 (0.79)	0.379	3.82 (0.82)	3.77 (0.92)	0.486
**Role conflict (1–5) **↓	3.35 (0.82)	3.46 (0.77)	0.012*	3.42 (0.69)	3.36 (0.77)	0.465
**Collaboration within unit (1–5) **↑	3.77 (0.72)	3.87 (0.69)	0.022*	3.77 (0.75)	3.66 (0.82)	0.069
**Collaboration between units (1–5) **↑	2.95 (0.72)	3.03 (0.71)	0.102	2.95 (0.82)	2.94 (0.66)	0.900
**Sleep quality (1–6) **↑	4.24 (1.10)	4.13 (1.05)	0.048*	4.18 (1.02)	4.25 (1.10)	0.391
**Restoration from sleep (1–6) **↑	4.16 (1.11)	4.20 (1.07)	0.485	4.31 (1.10)	4.20 (1.27)	0.163
**Relational justice (1–5) **↑	2.71 (0.97)	2.82 (0.93)	0.073	2.88 (0.82)	2.82 (0.84)	0.422
**Attitudes towards relocation (1–10) **↑	6.60 (2.93)	N/A	N/A	7.28 (2.56)	N/A	N/A
**Trust in management (1–5) **↑	3.52 (0.93)	3.66 (1.00)	0.030*	3.32 (0.98)	3.56 (0.99)	0.009*

Mean values, standard deviation (SD), *p*-values for the difference in mean values tested with paired sample t-test

Attitudes towards relocation was only measured at baseline

### Work environment problems and self-reported production loss at baseline and follow-up

At baseline, 51% of the respondents in organisation A and 57% of the respondents in organisation B had experienced work environment problems during the last 7 days. After relocation, 67% of the respondents in organisation A and 68% of the respondents in organisation B had experienced work environment problems during the last 7 days (Table 4). When analysing the impact of these work environment problems, self-reported production loss, the mean values were slightly higher at follow-up compared to baseline, however the group mean values did not differ significantly.

**Table 4:** Number of respondents reporting work environment problems during the last 7 days at baseline and at follow-up. Self-reported production loss due to work environment problems

		Organisation A			Organisation B		
		*N* (%)	Mean	SD	*N* (%)	Mean	SD
**Baseline**	**Work environment problems (Yes/No)**	140 (51)			62 (57)		
	**Self-reported production loss (0–10)**		3.63	2.17		3.10	2.22
**Follow-up**	**Work environment problems (Yes/No)**	185 (67)			74 (68)		
	**Self-reported production loss (0–10)**		4.01	2.38		4.34	2.60

Number of respondents (*N*) and percentage of respondents (%) reporting work environment problems during the last 7 days. Mean value and standard deviation (SD) for self-reported production loss. Rating scale ranging from 0 = The work environment problems had no effect on my work, to 10 = The work environment problems completely prevented me from working

### Associations between psychosocial factors and work environment problems– cross sectional analyses

At baseline, in Organisation A, significant associations were seen between all psychosocial factors (except quantitative demands) as well as sleep disturbances and sleep quality and experiencing work environment problems the last 7 days. The same pattern was seen after the implementation of the open office solutions, indicating that the same psychosocial factors were of importance for experiencing work environment problems both at baseline and at follow-up (See Appendix A, Table A3 and Table A4). The pattern was the same for organisation B.

### Associations between psychosocial factors and self-reported production loss due to work environmental problems– longitudinal analyses

When analysing the impact of baseline psychosocial factors on self-reported production loss due to work environment problems at follow-up, several significant associations were observed, and these associations varied between the organisations (see Table 5). Collaboration within units emerged as a significant factor in both organisations, where a more favourable collaboration was seen as a protective factor, OR 0.65; 95%CI 0.46–0.93 (Organisation A) and OR 0.50; 95%CI 0.29–0.86 (Organisation B), suggesting its importance. The results were more pronounced for Organisation B, where control of decisions (OR 0.42; 95%CI 0.25–0.69), collaboration between units (OR 0.50; 95%CI 0.30–0.86), and all three factors related to leadership: support from superiors (OR 0.54; 95%CI 0.35–0.83), relational justice (OR 0.55; 95%CI 0.33–0.90), and trust in management (OR 0.59; 95%CI 0.38–0.91). The direction of the associations for these variables were the same in Organisation A, but the results did not reach statistical significance. The reverse was seen for quantitative demands (OR 1.39; 95%CI 1.04–1.86) restoration from sleep (OR 0.77; 95%CI 0.62–0.95), and attitudes towards relocation (OR 0.89; 95%CI 0.82–0.97) where statistically significant associations were found solely in Organisation A.

**Table 5:** The individual association between different psychosocial factors at baseline, and self-reported production loss due to work environment problems at follow-up

	Organisation A		Organisation B	
	Odds Ratio	95%CI	Odds Ratio	95%CI
**Quantitative demands**	1.39*	1.04–1.86	1.31	0.81–2.13
**Control of decisions**	0.96	0.69–1.32	0.42*	0.25–0.69
**Support from superior**	0.94	0.74–1.21	0.54*	0.35–0.83
**Support from co-worker**	0.96	0.71–1.30	0.77	0.48–1.24
**Role clarity**	0.85	0.64–1.14	0.76	0.48–1.21
**Role conflict**	0.75	0.55–1.01	0.77	0.43–1.37
**Collaboration within unit**	0.65*	0.46–0.93	0.50*	0.29–0.86
**Collaboration between units**	1.01	0.73–1.42	0.50*	0.30–0.86
**Sleep quality**	0.91	0.73–1.12	0.98	0.65–1.48
**Restoration from sleep**	0.77*	0.62–0.95	0.71	0.48–1.03
**Relational justice**	0.89	0.69–1.14	0.55*	0.33–0.90
**Attitudes towards relocation**	0.89*	0.82–0.97	0.89	0.76–1.03
**Trust in management**	0.89	0.68–1.17	0.59*	0.38–0.91

Odds ratios and 95% confidence intervals (95% CI). Adjusted for age, gender, and self-reported production loss due to work environment problems at baseline. Stratified by organisation

### The influence of designated office types on psychosocial factors and work environment problems

At baseline, all respondents (in both organisations) were working either in cell offices or in shared rooms with 2–4 co-workers (Table 6). After relocation and implementation of the new office solutions, approximately 67% of the participants in Organisation A were designated to open plan offices or flex offices/activity-based offices. The corresponding number for Organisation B was 84%.

**Table 6:** Descriptive statistics of designated office types at baseline and at follow-up. Stratified by organisation

	Organisation A		Organisation B	
Office type	Baseline *N* (%)	Follow-up *N* (%)	Baseline *N* (%)	Follow-up *N* (%)
**Cell office**	121 (46)	46 (17)	62 (58)	12 (11)
**Shared office (2-4p)**	144 (54)	45 (16)	44 (42)	5 (5)
**Open plan office (> 4p)**	-	124 (45)	-	77 (72)
**Flex/Activity-based office**	-	59 (22)	-	13 (12)

Number of respondents (*N*) and percentage of respondents (%). Stratified by organisation

Before relocation the office types open plan office, and flex office/activity-based office did not exist within either of the organisations

### Psychosocial factors and designated office types

The analyses investigating whether exposure to psychosocial factors at baseline varied based on office type revealed that, in Organisation A, respondents in cell offices consistently reported a more adverse psychosocial work environment at baseline. This included higher quantitative demands, lower support from superiors and co-workers, stronger role conflict, weaker relational justice, and less trust in management, although the differences were relatively modest (see Table 7). In contrast, no such pattern was evident in Organisation B. Attitudes toward relocation did not significantly differ between cell offices and shared offices, with both organisations expressing positive sentiments toward relocation (on a scale of 1–10, where 10 represents the most positive attitude). In Organisation A, the mean value was 6.33 (SD 3.05) for cell offices and 6.83 (SD 2.81) for shared offices. In Organisation B, the mean value was 6.91 (SD 2.79) for cell offices and 7.78 (SD 2.03) for shared offices.

At follow-up in both organisations, the only exposure that differed between designated office types was the control of decisions. Participants in flex or activity-based offices reported the highest level of control of decisions compared to those in cell offices, shared offices, and traditional open-plan offices. Additionally, in Organisation A, exposure to quantitative demands differed between office types, with participants in cell offices reporting the highest level of quantitative demands, while participants in traditional open-plan offices reported the lowest level. No differences in other exposures were observed between office types (see Appendix A, Table A5).

**Table 7:** Psychosocial factors at baseline. Stratified by organisation and designated office type; cell office or shared office (2–4 p). In the Table, the direction of the question is indicated by an arrow, where ↓ = the lower the value the more positive the work environment is perceived. Correspondingly, ↑ (Higher value) indicates that the higher the value, the more positive the work environment is perceived

		Organisation A					Organisation B				
	Office types*	*N*	Mean	SD	*P*-value	Cohen’s d	*N*	Mean	SD	*P*-value	Cohen’s d
**Quantitative demands (1–5) **↓	CELL	119	3.49	0.75	0.000*	0.598	60	3.42	0.81	0.031*	0.435
	SHARED	141	3.04	0.76			44	3.09	0.72		
**Control of decisions (1–5) **↑	Cell	118	3.17	0.73	0.772	0.036	61	3.26	0.79	0.592	0.106
	Shared	144	3.14	0.72			44	3.34	0.63		
**Support from superior (1–5) **↑	Cell	119	3.57	1.07	0.008*	0.332	61	3.85	1.01	0.932	0.017
	Shared	143	3.91	0.96			44	3.86	0.96		
**Support from co-worker (1–5) **↑	Cell	120	4.05	0.92	0.000*	0.476	61	4.09	0.87	0.078	0.353
	Shared	143	4.42	0.63			44	4.36	0.62		
**Role clarity (1–5) **↑	Cell	118	3.73	0.86	0.070	0.227	60	3.86	0.85	0.576	0.112
	Shared	142	3.93	0.88			43	3.77	0.82		
**Role conflict (1–5) **↓	Cell	118	3.17	0.85	0.001*	0.417	61	3.34	0.74	0.177	0.269
	Shared	140	3.50	0.76			44	3.53	0.62		
**Collaboration within unit (1–5) **↑	Cell	120	3.69	0.76	0.131	0.188	60	3.88	0.71	0.166	0.277
	Shared	143	3.82	0.68			44	3.67	0.78		
**Collaboration between units (1–5) **↑	Cell	118	2.88	0.78	0.163	0.175	61	3.02	0.86	0.448	0.154
	Shared	141	3.01	0.66			41	2.89	0.78		
**Sleep quality (1–6) **↑	Cell	120	4.22	1.14	0.784	0.034	62	4.02	1.05	0.053	0.388
	Shared	140	4.26	1.07			43	4.42	0.97		
**Restoration from sleep (1–6) **↑	Cell	120	4.08	1.17	0.327	0.121	61	4.24	1.12	0.369	0.178
	Shared	140	4.22	1.09			44	4.43	1.09		
**Relational justice (1–5) **↑	Cell	120	2.49	1.05	< 0.001*	0.453	62	2.84	0.83	0.809	0.048
	Shared	144	2.91	0.84			44	2.88	0.85		
**Attitudes towards relocation (1–10) **↑	Cell	112	6.33	3.05	0.193	0.170	56	6.91	2.79	0.099	0.345
	Shared	123	6.83	2.81			40	7.78	2.03		
**Trust in management (1–5) **↑	Cell	120	3.38	1.01	0.030*	0.270	62	3.31	0.95	0.952	0.012
	Shared	144	3.63	0.84			44	3.32	1.05		

Number of respondents (*N*), Mean values (Mean), and standard deviation (SD). *P*-values for the difference in mean values tested with independent sample t-test. Effect size estimated with Cohen’s d* Before relocation the office types open plan office, and flex office/activity-based office did not exist within either of the organisations

At baseline, the proportions experiencing work environment problems during the last 7 days did not differ significantly between the office types cell office (54% in Organisation A and 58% in Organisation B) and shared office (51% in Organisation A and 52& in Organisation B) (Table 8). At follow-up (after relocation), the results showed that the highest proportion of respondents experiencing work environment problems in Organisation A was seen in the office type open plan office (72%). In organisation B, the majority of respondents experiencing work environment problem were seen in the office types shared office (100%) and open plan office (70%).

**Table 8:** At baseline and at follow-up, experienced work environment problems during the last 7 days. Stratified by organisation and designated office type

Office type	Organisation A		Organisation B	
	Baseline *N* (%)	Follow-up *N* (%)	Baseline *N* (%)	Follow-up *N* (%)
**Cell office**	65 (54)	28 (61)	36 (58)	7 (58)
**Shared office (2-4p)**	74 (51)	31 (69)	23 (52)	5 (100)
**Open plan office (> 4p)**	-	89 (72)	-	53 (70)
**Flex/Activity-based office**	-	35 (59)	-	7 (54)

Number of respondents (*N*) and percentage of respondents (%). Stratified by organisation

Before relocation the office types open plan office, and flex office/activity-based office did not exist within either of the organisations

## Discussion

The objective of the study was to explore potential associations between pre-existing psychosocial factors before implementation of open plan office solutions, and self-reported production loss due to work environment problems after the implementation. Regarding the investigated organisations in the present study, the implementation of new office solutions was initiated by the fact that both the organisations were to relocate into new premises. The primary driving forces were finance and co-locating different parts of the organisation into modern adapted premises that could house all departments in the organisation in a flexible solution. However, although those were the officially communicated reasons for the relocations, the findings in the present study also reveals that there was a high percentage of employees who, at baseline, reported work environment problems (51% in Organisation A, and 57% in Organisation B) during the last seven days, and that these work environment problems also affected their productivity. According to earlier research, cell offices are often rated as the most wanted office design, where employees experience less problems, but in the present study work environment problems were found to be somewhat worse in cell offices compared to shared offices (at baseline) [5, 44].

Regarding different psychosocial factors, although no clear patterns were revealed, some differences between employees working in cell office or employees working in shared offices were seen. In Organisation A, employees working in cell office experienced higher quantitative demands and lower support (from both superior and co-workers). Further, they experienced the relational justice in the organisation as worse and had a lower trust in management. The findings that support from manager, support from co-workers and relational justice all were rated higher among employees in shared offices compared to employees in cell-offices might be related to the employee’s experiences of workload and work demands, which might be higher if you got your own office space, or the fact that several managers work in a cell office than other employees do. In organisation B, there was a tendency towards the same results regarding quantitative demands and support from co-workers, but exposures such as support from superior, relational justice and trust in management did not differ based on office type.

The associations between psychosocial factors, the experience of work environment problems, and productivity loss exhibited variations between the organisations. The diverse outcomes appear to be influenced by the distinct dynamics of each complex organisation. The question arises: what conclusions can be drawn, and which psychosocial factors can be interpreted as influencing the perception of the work environment one year after implementation? In summary, factors related to leadership and collaboration emerge as prominent in the overall results, all of which are strongly associated with fostering a healthy organisational environment [18].

The results at baseline shows that employees that rated their psychosocial work environment as worse also experienced over-all work environment problems to a higher degree. This was consistent in both organisations and for almost all psychosocial factors.

Even if the relocation was not initiated by work environment issues, it can be expected that the relocation could affect the exposure to different psychosocial factors as well as the over-all experience of work environment problems and self-reported production loss in one direction or the other. A common way to evaluate the implementation of new office solutions is to compare exposure to different psychosocial factors at baseline with exposure to the same factors at follow-up. Previous studies have found that the psychosocial work environment is affected in several ways, and that open plan office solutions, poses challenges with regards to i.e. concentration, distraction and noise disturbance [5–7]. In the present study some statistically significant differences with regards to changes of exposure to different psychosocial factors were found at follow-up, but the differences were small, and difficult to interpret. However, the variable “trust in management” showed a more evident positive increase in both organisations (even if reaching significance only in Organisation A), which could indicate that the management in both organisations handled the implementation of the new office solutions in a satisfactory manner. This is further validated by the fact that the employees in both organisations to a large extent, in both cell offices and shared offices, had a positive attitude towards the relocation at baseline, suggesting that the organisations were successful in promoting a positive attitude towards the office change. This might have been influenced by support and clarity from management and opportunities for participation early on in the implementation.

Although the trust in management had improved at follow-up, there was an increased proportion of employees in both organisations who experienced work environment problems (67% in Organisation A and 68% in Organisation B). The highest prevalence of perceived work environment problems was seen in the open plan offices compared to cell offices and flex/activity-based offices. This is in line with previous studies which have shown that the traditional open plan offices are often perceived to be more problematic. Earlier research within this area has indicated that employees in small and medium sized open plan offices reported the most ill-health, followed by employees in shared rooms and large open plan offices [45]. In a recent Swedish study, the employees in open plan offices reported a decrease in perceived productivity while the cell office group’s productivity rating remained unchanged [13]. The general perception that an activity-based office is superior to traditional open-plan offices in terms of health and productivity arises from several factors that address the limitations of conventional office designs. For instance, activity-based offices are designed to offer greater flexibility, allowing employees to choose workspaces that align with their specific needs, be it for collaboration and informal meetings, remote work, or quiet zones to mitigate high noise levels and distractions. According to current research, the anticipated improvements and effectiveness of an activity-based office vary, and companies should tailor their office design to their unique circumstances and workforce requirements [5–7].

Further, from a business perspective, the British company Leesman LTD in 2019 published a bench marketing report which was an effectiveness evaluation of how activity-based offices perform and support employees in comparison with other office designs [46]. One conclusion in their latest report was that one in five new work places fails to meet employee needs, which shows that there is no office design that fits all. Another conclusion was that one in three did not agree that their new workplace allowed them to be productive. Research this far seems to agree to the fact that open plan office solutions, and more specifically activity-based offices, do not suit all kinds of organisations/businesses, all work tasks or all individuals [7].

Working life involves constant challenges that need to be met and implementing a major change in an organisation is burdensome for those involved. A supportive psychosocial work environment can enable an organisation to have greater resilience and preparedness for change. A relocation and implementation of a new office design also involves organisational changes as how the work is performed, and the implementation process has shown to be of great importance for a successful outcome [6, 13]. Hence it was of interest to investigate the impact of baseline psychosocial factors on work environment problems and related production loss at follow-up.

The results show that although the tendencies concerning associations between different psychosocial factors and production loss are in the same direction for both organisations, the statistically significant results varies between the organisations. There may be several explanations for this, one being lack of statistical power. Another a highly plausible explanation may lie in the fact that there may be structural differences between the two organisations, with different contexts, where some psychosocial factors may be of different importance in the two organisations. An example of this may be the illustrated by results concerning the importance of collaboration between units, which in Organisation B, was significantly associated with production loss (OR = 0.50; 95%CI 0.30–0.86), compared to in Organisation A (OR = 1.01; 95%CI 0.73–1.42). These results could illustrate that in Organisation B, collaboration between units is of great importance to be able to successfully perform different work tasks, whilst Organisation A may be an organisation with more independent and self-managing departments. This may also serve as a potential explanation as to why three variables concerning management and leadership (support from superior, relational justice and trust in management) showed significant associations with production loss in Organisation B, associations that were not seen clearly in Organisation A, hypothesising that Organisation A is constituted by self-governing and autonomous units, more dependent of self-leadership and self-control. This is in line with earlier research about different kinds of self-managing organisations. The most obvious characteristic is that the power to make more comprehensive decisions is delegated and employees are empowered to make decisions themselves concerning their departments [47].

Another hypothesis is that the differences regarding the associations concerning leadership and production loss might be due to a difference in how the implementation process was carried out in the two organisations. Although the somewhat divergent results between the organisations, it is of interest to discuss possible interactions between some of the more prominent results.

Relational justice was a factor that in Organisation B showed to have great importance, and there was a tendency towards the same result in organisation A. It is noteworthy that factors connected to the concept of relational justice, such as leadership (support from superior and relational justice) and collaboration (within and between units) also seem to be important factors before an organisational change in order to prevent work environmental related self-reported production loss after the change. Relational justice has been shown to contribute to creating a more positive work environment where employees have high satisfaction and feels motivated to perform at work [48]. When employees are being treated fairly by management, their motivation to perform at work and to contribute to the success of the organisation increases. Further, earlier studies have identified how the brain reacts to social injustice. When experience of justice, the reaction is the same as in the experience of reward, and in the case of injustice, it is instead experienced as a threat [49]. The results in the present study can be interpreted as having a stronger relational justice in an organisation may benefit the organisation with regards to coping with work environment challenges in office implementations.

Further, earlier research has shown that good relational justice is signified by a work environment where there is a high degree of participation and respectfulness by colleagues and managers, where employees are more likely to feel comfortable expressing themselves and communicating openly and honestly [27, 50]. This supports the findings in the present study that good communication, in terms of collaboration between colleagues decreases the risk of experiencing production loss due to work environment problems.

Relational justice can also be considered to be closely related to trust in management. The results in the present study showed that having trust in management decreased the risk of production loss. Trust in management can be said to affect employee engagement, motivation and work performance. If employees do not have confidence in the leadership, it may lead to disengagement and demotivation at work, which in turn negatively affects work performance [51].

Sleep disturbances is an important health indicator for the organisation, a poor work environment leads to increased stress, worry and anxiety, which in turn can make it harder to fall asleep at night and cause sleep disorders. Promoting good sleep in the workplace can lead to increased productivity. A review (Linton et al., 2014) showed that the psychosocial factors such as social support at work, control, and relational justice were related to fewer sleep disturbances, while high work demands, job strain, bullying, and effort-reward imbalance were related to more future sleep disturbances [23]. The psychosocial factors have an impact on sleep disturbances. In the present study the index “restoration from sleep” seems to be stronger associated with self-reported production loss, compared to sleep quality. This is in line with previous studies that points out sleep as a powerful recovery mechanism, which affects virtually all biological and psychological functions in the body [52]. Stress is one of the most common causes of sleep disturbances, but stress is also considered multi-factorial, and the sleep disturbances reported by the participants may have been influenced by other factors not investigated in the present study, such as impaired general health, physical problems, drug-induced sleeping problems or family related problems.

### Strengths and limitations

The design of the analyses was collaboratively developed with a statistician, carefully aligning it with both the questionnaire’s structure and the study’s objectives.

Production loss was selected as the main outcome variable. Productivity can be seen as a tangible metric for the organisation’s performance, offering a more concrete measure than the well-being and health of employees. This choice of productivity as a measure is likely to attract the interest of employers in engaging with the study’s results. Productivity is a broad concept, and different aspects of productivity are commonly used within the industry sector such as output per labour hour, lead time and cycle time, scrap and rework rates and production yield. However, productivity may be more difficult to measure in the public sector and among professions in administration and various types of support functions. Productivity and production loss are also influenced by factors such as employee engagement and exposure to different work environment factors [53]. In the present study, productivity was measured using questions developed by Swedish researchers in health economics [22, 33]. This measure of work environment-related production loss has been validated and evaluated concerning the significant difference in the proportion of explained variation of production loss [22]. Further, convergent validity and test-retest reliability has also been tested [32]. The conclusions drawn from these studies are that the measure can be utilized to identify production loss attributable to work environment problems and can serve as an outcome measure when evaluating the impact of organisational interventions [33].

It is worth noticing that the study primarily focused on work-related factors rather than general health. The latter is undeniably significant for workplace well-being and productivity and previous research on production loss has also found that the associations between psychosocial factors and self-reported work related production loss can be is mediated by employee health [33].

As with any scientific paper, the present study has both some strengths and some limitations. A strength in this study is the longitudinal design with two measuring points. However, this can paradoxically enough, be considered a limitation in all organisational research, as it is well known that there is a difficulty to control what happens in the organisation between baseline and follow-up. It is not unusual that several changes are taken place at the same time, which can make it difficult to attribute the results in a study to the particular phenomena studied. In the present study, the two organisations were closely followed using both questionnaires and interviews and the researchers regularly took part in the project groups meetings. To the knowledge of the authors, no major structural changes were implemented during the relocation and follow-up period. Still, other day-to-day factors may influence workplace dynamics and can have affected the results in the present study. Factors such as new workflows and new ways of working, digitization or changes in workload, physical factors concerning the office, office geographical location (closeness to green areas, recreational center, shopping) and differences among employees preferences concerning office design.

Differences in the two organizations’ implementation processes might also have affected the results, and the implementation process will be explored in a forthcoming qualitative study on the same two organizations.

The follow-up assessment was made 12 months after the relocation to the new premises, a relatively short time. There is a possibility that the current results may be reactions to the actual relocation process [54]. In future research, to be able to assess long-term effects a longer follow-up period, or several follow-up assessments should be considered.

Another limitation is that the baseline measurement points differ between the two organisations, occurring six months ahead of relocation in Organisation A and eight months before relocation in Organisation B. The original plan was to synchronize these measurement points, but it proved unfeasible due to circumstances in the implementation processes. We believe that this difference has had no impact on the results. The measurements were conducted well in advance to prevent the organisation from being already influenced by the upcoming change process, and not too far ahead to accurately capture the current situation. Both organisations’ measurement points meet these criteria. However, the time point for the follow-up measurement, after relocation, was maintained within the same time period.

A strength is that the study included two organisations with different conditions and different contexts, but that the organisations were comparable as both being within the public sector and similar in terms of background factors (gender, age, education). By performing the analyses separately for the two organisations, it was possible to validate the findings by comparing the results in the two organisations. Another strength in the study is the data collection allowed for the use of statistical inference with paired analyses, which can be considered the preferable choice in order to investigate complex relationships, and is advantageous with regards to statistical power, for example in terms of controlling for confounding variables. As an extra precaution, all participants in the analysis had the same type of position (employee/manager) at both measurement points. Further, the response rate in the present study may be considered to be relatively high. Although what constitutes an acceptable response rate depends on the context and the specific characteristics of the study, some earlier studies have mentioned response rates above 65% as fairly acceptable [55–57]. 

As for the drop-out analysis, a slightly higher drop-out was seen with regards to sex, and age. However, the differences between responders and non-responders were small, and it is unlikely that it could have affected the results. No drop-out analysis could be done of the baseline measurements.

The present study focused on psychosocial factors, and to investigate the prevalence of work environment problems a rather general definition of work environment problems was used. A limitation in the analyses is that, since the questionnaire did not include any questions regarding physical work environment exposures, there was no possibility to adjust for this in the analyses. Hence, the associations found in the present study, may be diluted due to these shortcomings.

Earlier research has shown that psychosocial factors may differ depending on hierarchal position in an organisation, for example managers may more often experience high demand, problems in relation to unclear goals, and an imbalance in terms of responsibility and authority [58]. In the present study there was not enough statistical power to do stratified analyses on managers. Another strength in the study is the used of well-established questions concerning psychosocial factors that have been extensively used in previous research. These factors are considered stable over time and resilient to societal changes, such as economic fluctuations and the Covid-19 pandemic. Covid-19 has had a major impact on working life, both during and after the pandemic, especially in terms of the increased possibility for office workers to work remotely, which has led to other needs concerning office designs and working dynamics [59–61]. The possibility for teleworking was not investigated in the present study, as it was almost non-existent in the Swedish public sector during the time for relocation in 2016–2017.

As for generalisability, the two participating management/administrative departments can be considered to be representative for similar departments within public organisations. However, such large organisations consists of several types of departments and, depending on work tasks and focus, the results may not be transferrable to the entire organisations.

### Future research

The present study aimed to investigate the potential associations between pre-existing psychosocial factors before implementation of open plan office solutions, and the results points towards the importance of taking great consideration into preparing the organisation by working actively with a number of psychosocial factors, mainly related to relational justice, leadership and collaboration. However, the results also show that what factors that are of importance to put emphasis on, may be closely related to the organisational structure and the work tasks at hand. These findings are something that needs to be more deeply investigated in future research.

In any implementation process, it is of importance that the organisation is prepared for change [62]. The results in the present study highlights the importance of creating a positive psychosocial work environment in the preparation for change. This needs to do be done in a systematic and persistent manner, and in a continuous process with recurring activities as a part of the systematic work environment management [63, 64]. It might feel insurmountable to create a sustainable and health promoting work environment, and difficult for the employer to know where and how. To aid with the systematic work environment management, the employer can engage an external work environment expertise such as an Occupational Health Service (OHS) provider. Facilitating factors for such collaborations has been investigated in earlier studies [65, 66]. However, how the occupational health service can aid in the preparation for change is something that needs to be investigated in the future.

Earlier research has found that in recently changed organisations, a transformational leadership is beneficial for worker commitment and comprehension about the assignment, in other words, the leadership has an impact on the employees’ health and productivity [67]. The new open plan office design affects the leaderships behaviour. This implies that organisations need to provide managers with prerequisites to be able to keep up with behaviours that support employees’ performance and health when office designs and ways of working are changed [11]. Managers have an important role and a great opportunity to influence the office change in the right direction. As for the possibilities to explore this in the present study, the questionnaire did contain some questions about leadership. However, these questions have solely been analysed as for potential associations with productivity loss. In the larger overarching project, qualitative data concerning the relocation has been collected through focus groups interviews, which in the future will be analysed in depth regarding the influence of leadership in implementation of new office design.

The concept “New ways of working” have been influencing the working life, to facilitate development and adept to new opportunities in digitalisation [3, 4]. After the Covid − 19 pandemic the trend of remote work, with flexibility and self-leadership has become even more stronger. This poses a great challenge in today’s working life, and office research, needs to more clearly explore the office best usability and significance in creating a positive work environment.

A well-designed modern office can create a positive work environment, fostering collaboration, creativity, and employee well-being. On the other hand, poorly designed offices, noise, and overcrowding can contribute to stress, anxiety, and other health problems. This study delves into the experiences of employees in their workplace, a space where a significant amount of time is invested. The importance of cultivating a positive work environment is underscored, as it directly contributes to our overall well-being. Conversely, our well-being during leisure time can impact how we feel at work [68, 69]. Striking a healthy balance between work and leisure is crucial for fostering a sustainable and enduring professional [70].

## Conclusions

The present study, exploring two organisations within the same sector, contributes with the knowledge that change always takes place in a unique context for each specific organisation. Several pre-existing psychosocial factors before implementation of new office designs were found to be associated with self-reported production loss due to work environment problems at follow-up. Differences were seen between the organisations and the statistical strengths of the associations varied. However, the tendencies concerning the associations between different psychosocial factors and production loss are in the same direction for both organisations.

Collaboration within units emerged as a significant factor in both organisations, where a more favourable collaboration was seen as a protective factor, suggesting its importance.

The results were more pronounced in organisation B, where all three factors related to leadership: support from superiors, relational justice, and trust in management seemed to be of importance. The direction of the associations for these variables were the same in Organisation A, but the results did not reach statistical significance. As for the reverse, Quantitative demands emerged as a significant factor in organisation A, but in organisation B, however, the results were not statistically significant. The mechanisms influencing work environment factors are interconnected and complex, and there is a need for qualitative studies exploring the importance of for instance organisational culture and structure, characteristics and content of work tasks, and individual characteristics in the implementation of new office designs.

## Electronic supplementary material

Below is the link to the electronic supplementary material.

Supplementary Material 1

## Data Availability

The datasets generated and analyzed during the current study are not publicly available due to confidentiality reasons. Data can be made available upon reasonable request from the corresponding author.
