# Supplementary material for: The influence of psychosocial factors on productivity when implementing new office designs - a longitudinal explorative study in the Swedish public sector
Source: BMC Public Health. 2025 May 23;25:1894. doi: 10.1186/s12889-025-22953-4 (PMC12100884; doi:10.1186/s12889-025-22953-4)
Supplement: Supplementary file 1 — Supplementary Material 1 [file 12889_2025_22953_MOESM1_ESM.docx]

## Appendix A

**Table A1. Indices used in the questionnaire. Cronbach’s alpha values and Cronbach’s alpha if item deleted.**

| **INDEX** | **CRONBACH'S ALPHA** | **ITEMS** | **CRONBACH'S ALPHA IF ITEM DELETED** |
| --- | --- | --- | --- |
| **Quantitative demands** | 0.832 | Is your work load irregular so that the work piles up? | 0.803 |
|  |  | Do you have to work overtime? | 0.796 |
|  |  | Is it necessary to work at a rapid pace? | 0.775 |
|  |  | Do you have too much to do? | 0.777 |
| **Control of decisions** | 0.720 | If there are alternative methods for doing your work, can you choose which method to use? | 0.688 |
|  |  | Can you influence the amount of work assigned to you? | 0.680 |
|  |  | Can you influence decisions concerning the persons you will need to collaborate with? | 0.645 |
|  |  | Can you influence decisions that are important for your work? | 0.617 |
| **Support from superior** | 0.871 | If needed, can you get support and help with your work from your immediate superior? | 0.783 |
|  |  | If needed, is your immediate willing to listen to your task-related problems? | 0.793 |
|  |  | Are your work achievements appreciated by our immediate superior? | 0.876 |
| **Support from co-workers** | 0.821 | If needed, can you get support and help with your work from your co-workers? | n/a |
|  |  | If needed, are your co-workers willing to listen to your work-related problems? | n/a |
| **Role clarity** | 0.839 | Have clear, planned goals and objectives been defined for you? | 0.802 |
|  |  | Do you know what your responsibilities are? | 0.769 |
|  |  | Do you know exactly what is expected of you at work? | 0.762 |
| **Role conflict** | 0.708 | Do you have to do things that you feel should be done differently? | 0.740 |
|  |  | Are you given assignments without adequate resources to complete them? | 0.505 |
|  |  | Do you receive incompatible requests from two or more people? | 0.561 |
| **Collaboration within unit** | 0.838 | How is the productivity within the team? | 0.812 |
|  |  | How does intra-team cooperation work? | 0.751 |
|  |  | How well do new ideas spread within the team/unit? | 0.815 |
|  |  | How well does work-related communication function between the colleagues in your team/unit? | 0.797 |
| **Collaboration between units** | 0.794 | How does cooperation with other teams/units work? | 0.744 |
|  |  | How well do new ideas spread to other teams/units? | 0.708 |
|  |  | How do you perceive the spreading of information between units all in all? | 0.708 |
| **Sleep quality** | 0.849 | difficulty falling asleep | 0.840 |
|  |  | waking up with difficulty going back to sleep | 0.769 |
|  |  | waking up too early | 0.835 |
|  |  | having restless/disturbed sleep | 0.784 |
| **Restoration from sleep** | 0.812 | difficulty waking up | 0.700 |
|  |  | waking up feeling unrested | 0.848 |
|  |  | waking up fatigued | 0.651 |
| **Relational Justice** | 0.873 | Your supervisor considers your viewpoints. | 0.846 |
|  |  | Your supervisor is able to suppress personal biases. | 0.852 |
|  |  | Your supervisor provides you with timely feedback about the decisions and their implications. | 0.858 |
|  |  | Your supervisor treats you with kindness and consideration. | 0.847 |
|  |  | Your supervisor shows concern for your rights as an employee. | 0.857 |
|  |  | Your supervisor takes steps to deal with you in a truthful manner. | 0.850 |

**Table A2. Responders and non-responders at follow-up. Based on the respondents at baseline. Stratified by organisation. Number of respondents (n), age (years), employment > 3 years, and education level at baseline.**

|  | **ORGANISATION A** | | **ORGANISATION B** | |
| --- | --- | --- | --- | --- |
|  | ***Responders*** | ***Non-responders*** | ***Responders*** | ***Non-responders*** |
|  | ***N (%)*** | ***N (%)*** | ***N (%)*** | ***N (%)*** |
| **N women/men** | 187/98 (66/34) | 81/52 (61/39) | 77/36 (68/32) | 51/27 (65/35) |
| **Employment >3 years (baseline)** | 190 (70) | 89 (60) | 72 (62) | 46 (68) |
| **Age (Baseline)** |  |  |  |  |
| < 40 years | 61 (20) | 38 (29) | 25 (24) | 22 (29) |
| 41-50 years | 109 (37) | 48 (36) | 36 (30) | 27 (35) |
| 51-60 years | 99 (35) | 29 (22) | 41 (35) | 12 (16) |
| >60 years | 14 (8) | 17 (13) | 11 (11) | 16 (21) |
| **Education** |  |  |  |  |
| High school level | 48 (14) | 15 (11) | 20 (18) | 9 (14) |
| University level | 223 (86) | 114 (86) | 93 (82) | 65 (86) |
| Number of responders/non-responders (N) and percentage of responders/non-responders (%), age at baseline, and education level at baseline. | | | | |

Table A3. Psychosocial work environment exposures at baseline. Stratified by organisation and experienced work environment problems during the last 7 days (Yes/No). In the table, the direction of the question is indicated by an arrow, where ↓ = the lower the value the more positive the work environment is perceived. Correspondingly, ↑ (Higher value) indicates that the higher the value, the more positive the work environment is perceived.

|  |  | **ORGANISATION A** | | | | | **ORGANISATION B** | | | | |
| --- | --- | --- | --- | --- | --- | --- | --- | --- | --- | --- | --- |
|  | **Work environment problems** | **N** | **Mean** | **SD** | **p-value** | **Cohen’s d** | **N** | **Mean** | **SD** | **p-value** | **Cohen’s d** |
| **Quantitative demands (1-5) ↓** | YES | 138 | 3.32 | 0.83 | 0.113 | 0.197 | 60 | 3.30 | 0.78 | 0.547 | 0.118 |
|  | NO | 123 | 3.17 | 0.72 |  |  | 47 | 3.21 | 0.79 |  |  |
| **Control of decisions (1-5) ↑** | YES | 138 | 2.99 | 0.69 | 0.000* | 0.493 | 61 | 3.09 | 0.77 | 0.007* | 0.534 |
|  | NO | 126 | 3.34 | 0.72 |  |  | 47 | 3.48 | 0.64 |  |  |
| **Support from superior (1-5) ↑** | YES | 137 | 3.44 | 1.08 | 0.000* | 0.685 | 62 | 3.53 | 0.98 | 0.000* | 0.864 |
|  | NO | 125 | 4.10 | 0.84 |  |  | 46 | 4.31 | 0.78 |  |  |
| **Support from co-worker (1-5) ↑** | YES | 137 | 4.09 | 0.84 | 0.001* | 0.422 | 62 | 4.15 | 0.87 | 0.288 | 0.208 |
|  | NO | 127 | 4.42 | 0.72 |  |  | 46 | 4.32 | 0.64 |  |  |
| **Role clarity (1-5) ↑** | YES | 136 | 3.67 | 0.89 | 0.001* | 0.430 | 60 | 3.69 | 0.88 | 0.067 | 0.363 |
|  | NO | 125 | 4.04 | 0.81 |  |  | 46 | 3.99 | 0.73 |  |  |
| **Role conflict (1-5) ↓** | YES | 134 | 3.09 | 0.76 | 0.000* | 0.688 | 61 | 3.26 | 0.72 | 0.006* | 0.539 |
|  | NO | 125 | 3.63 | 0.79 |  |  | 47 | 3.62 | 0.60 |  |  |
| **Cooperation within unit (1-5) ↑** | YES | 137 | 3.52 | 0.73 | 0.000* | 0.759 | 60 | 3.62 | 0.76 | 0.018* | 0.470 |
|  | NO | 127 | 4.04 | 0.62 |  |  | 47 | 3.96 | 0.69 |  |  |
| **Cooperation between units (1-5) ↑** | YES | 135 | 2.79 | 0.76 | 0.000* | 0.509 | 58 | 2.76 | 0.83 | 0.006* | 0.546 |
|  | NO | 125 | 3.14 | 0.63 |  |  | 47 | 3.20 | 0.75 |  |  |
| **Sleep quality (1-6) ↑** | YES | 136 | 4.03 | 1.12 | 0.001* | 0.413 | 61 | 3.98 | 1.06 | 0.019* | 0.461 |
|  | NO | 125 | 4.48 | 1.04 |  |  | 47 | 4.44 | 0.91 |  |  |
| **Restoration from sleep (1-6) ↑** | YES | 139 | 3.89 | 1.12 | 0.000* | 0.521 | 62 | 4.04 | 1.23 | 0.003* | 0.599 |
|  | NO | 126 | 4.46 | 1.05 |  |  | 46 | 4.67 | 0.77 |  |  |
| **Relational justice (1-5) ↑** | YES | 138 | 2.41 | 1.03 | 0.000* | 0.724 | 62 | 2.65 | 0.88 | 0.002* | 0.626 |
|  | NO | 127 | 3.06 | 0.75 |  |  | 47 | 3.15 | 0.67 |  |  |
| **Trust in management (1-5) ↑** | YES | 138 | 3.36 | 0.93 | 0.002* | 0.380 | 62 | 3.00 | 1.04 | 0.000* | 0.791 |
|  | NO | 127 | 3.70 | 0.89 |  |  | 47 | 3.72 | 0.71 |  |  |
| Mean values, standard deviation (SD), p-values for the difference in mean values tested with independent sample t-test (equal variance assumed). Effect size estimated with Cohen’s d. | | | | | | | | | | | |

Table A4. Psychosocial work environment exposures at follow-up. Stratified by organisation and experienced work environment problems during the last 7 days (Yes/No). In the table, the direction of the question is indicated by an arrow, where ↓ = the lower the value the more positive the work environment is perceived. Correspondingly, ↑ (Higher value) indicates that the higher the value, the more positive the work environment is perceived.

|  |  | **ORGANISATION A** | | | | | **ORGANISATION B** | | | | |
| --- | --- | --- | --- | --- | --- | --- | --- | --- | --- | --- | --- |
|  | **Work environment problems** | **N** | **Mean** | **SD** | **p-value** | **Cohen’s d** | **N** | **Mean** | **SD** | **p-value** | **Cohen’s d** |
| **Quantitative demands (1-5) ↓** | YES | 184 | 3.19 | 0.83 | 0.072 | 0.233 | 72 | 3.23 | 0.71 | 0.755 | 0.065 |
|  | NO | 89 | 3.00 | 0.76 |  |  | 34 | 3.18 | 0.83 |  |  |
| **Control of decisions (1-5) ↑** | YES | 182 | 3.10 | 0.70 | 0.002* | 0.408 | 73 | 3.11 | 0.72 | 0.000* | 1.113 |
|  | NO | 90 | 3.39 | 0.73 |  |  | 35 | 3.86 | 0.55 |  |  |
| **Support from superior (1-5) ↑** | YES | 185 | 3.78 | 1.03 | 0.002* | 0.408 | 74 | 3.55 | 1.04 | 0.017* | 0.498 |
|  | NO | 90 | 4.17 | 0.81 |  |  | 35 | 4.03 | 0.72 |  |  |
| **Support from co-worker (1-5) ↑** | YES | 183 | 4.18 | 0.90 | 0.144 | 0.189 | 72 | 4.16 | 0.81 | 0.851 | 0.039 |
|  | NO | 90 | 4.33 | 0.66 |  |  | 35 | 4.13 | 0.79 |  |  |
| **Role clarity (1-5) ↑** | YES | 182 | 3.78 | 0.84 | 0.002* | 0.416 | 71 | 3.69 | 0.96 | 0.140 | 0.310 |
|  | NO | 88 | 4.11 | 0.68 |  |  | 34 | 3.97 | 0.81 |  |  |
| **Role conflict (1-5) ↓** | YES | 185 | 3.37 | 0.76 | 0.002* | 0.403 | 74 | 3.26 | 0.78 | 0.042* | 0.423 |
|  | NO | 90 | 3.67 | 0.74 |  |  | 35 | 3.58 | 0.70 |  |  |
| **Cooperation within unit (1-5) ↑** | YES | 183 | 3.73 | 0.73 | 0.000* | 0.664 | 73 | 3.50 | 0.88 | 0.004* | 0.597 |
|  | NO | 89 | 4.17 | 0.47 |  |  | 35 | 3.97 | 0.52 |  |  |
| **Cooperation between units (1-5) ↑** | YES | 182 | 2.94 | 0.73 | 0.001* | 0.426 | 74 | 2.79 | 0.68 | 0.001* | 0.692 |
|  | NO | 88 | 3.24 | 0.65 |  |  | 34 | 3.23 | 0.52 |  |  |
| **Sleep quality (1-6) ↑** | YES | 182 | 4.09 | 1.04 | 0.241 | 0.153 | 73 | 4.12 | 1.20 | 0.039* | 0.430 |
|  | NO | 88 | 4.25 | 1.06 |  |  | 35 | 4.59 | 0.80 |  |  |
| **Restoration from sleep (1-6) ↑** | YES | 185 | 4.16 | 1.06 | 0.339 | 0.124 | 74 | 4.01 | 1.36 | 0.017* | 0.495 |
|  | NO | 88 | 4.29 | 1.09 |  |  | 35 | 4.63 | 0.92 |  |  |
| **Relational justice (1-5) ↑** | YES | 184 | 2.74 | 0.96 | 0.004* | 0.375 | 73 | 2.69 | 0.92 | 0.020* | 0.487 |
|  | NO | 89 | 3.08 | 0.84 |  |  | 35 | 3.10 | 0.58 |  |  |
| **Trust in management (1-5) ↑** | YES | 184 | 3.51 | 0.99 | 0.000* | 0.547 | 73 | 3.37 | 1.07 | 0.004* | 0.599 |
|  | NO | 90 | 4.03 | 0.92 |  |  | 35 | 3.94 | 0.64 |  |  |
| Mean values, standard deviation (SD), p-values for the difference in mean values tested with independent sample t-test (equal variance assumed). Effect size estimated with Cohen’s d. | | | | | | | | | | | |

**Table A5. Psychosocial work environment exposures at follow-up. Stratified by organisation and designated office type. Number of respondents (N), Mean values (Mean), and standard deviation (SD). Difference in mean values tested with one-way ANOVA analyses and Kruskal–Wallis H tests.**

|  |  | **ORGANISATION A** | | | **ORGANISATION B** | | |
| --- | --- | --- | --- | --- | --- | --- | --- |
|  | **Office types*** | **N** | **Mean** | **SD** | **N** | **Mean** | **SD** |
| **Quantitative demands^A^** | CELL | 46 | 3.34 | 0.76 | 11 | 3.16 | 0.97 |
|  | SHARED | 44 | 3.14 | 0.92 | 5 | 2.50 | 0.92 |
|  | OPEN PLAN | 124 | 2.97 | 0.80 | 75 | 3.26 | 0.71 |
|  | FLEX/ABW | 58 | 3.32 | 0.72 | 13 | 3.27 | 0.70 |
| **Control of decisions^A,B^** | CELL | 45 | 3.19 | 0.83 | 12 | 3.42 | 0.74 |
|  | SHARED | 45 | 3.03 | 0.87 | 5 | 2.55 | 0.54 |
|  | OPEN PLAN | 123 | 3.14 | 0.64 | 77 | 3.31 | 0.73 |
|  | FLEX/ABW | 58 | 3.46 | 0.63 | 13 | 3.94 | 0.58 |
| **Support from superior** | CELL | 46 | 3.93 | 0.95 | 12 | 3.92 | 1.06 |
|  | SHARED | 45 | 3.72 | 1.19 | 5 | 2.73 | 1.14 |
|  | OPEN PLAN | 124 | 3.90 | 0.94 | 77 | 3.72 | 0.96 |
|  | FLEX/ABW | 59 | 4.06 | 0.91 | 13 | 3.77 | 0.88 |
| **Support from co-worker** | CELL | 46 | 4.08 | 0.91 | 12 | 4.08 | 0.70 |
|  | SHARED | 45 | 4.19 | 0.87 | 5 | 4.30 | 0.84 |
|  | OPEN PLAN | 123 | 4.32 | 0.73 | 75 | 4.20 | 0.81 |
|  | FLEX/ABW | 58 | 4.17 | 0.91 | 13 | 3.81 | 0.80 |
| **Role clarity** | CELL | 45 | 3.92 | 0.79 | 12 | 4.25 | 1.04 |
|  | SHARED | 45 | 3.96 | 0.88 | 4 | 3.42 | 1.62 |
|  | OPEN PLAN | 122 | 3.81 | 0.84 | 74 | 3.72 | 0.88 |
|  | FLEX/ABW | 57 | 3.98 | 0.67 | 13 | 3.77 | 0.82 |
| **Role conflict** | CELL | 46 | 3.41 | 0.85 | 12 | 3.53 | 0.80 |
|  | SHARED | 45 | 3.34 | 0.77 | 5 | 3.40 | 0.76 |
|  | OPEN PLAN | 124 | 3.56 | 0.76 | 77 | 3.30 | 0.80 |
|  | FLEX/ABW | 59 | 3.40 | 0.72 | 13 | 3.51 | 0.63 |
| **Collaboration within unit** | CELL | 46 | 3.95 | 0.64 | 12 | 3.77 | 1.00 |
|  | SHARED | 45 | 3.73 | 0.82 | 5 | 2.65 | 0.91 |
|  | OPEN PLAN | 123 | 3.85 | 0.67 | 76 | 3.69 | 0.80 |
|  | FLEX/ABW | 57 | 3.98 | 0.65 | 13 | 3.77 | 0.43 |
| **Collaboration between units** | CELL | 46 | 3.12 | 0.79 | 12 | 3.17 | 0.64 |
|  | SHARED | 45 | 3.01 | 0.85 | 5 | 2.60 | 0.55 |
|  | OPEN PLAN | 121 | 2.97 | 0.70 | 76 | 2.86 | 0.69 |
|  | FLEX/ABW | 57 | 3.11 | 0.58 | 13 | 3.20 | 0.50 |
| **Sleep quality** | CELL | 43 | 3.91 | 0.99 | 12 | 4.06 | 1.10 |
|  | SHARED | 45 | 4.29 | 0.95 | 5 | 3.95 | 1.97 |
|  | OPEN PLAN | 122 | 4.16 | 1.09 | 76 | 4.26 | 1.10 |
|  | FLEX/ABW | 59 | 4.15 | 1.06 | 13 | 4.73 | 0.77 |
| **Restoration from sleep** | CELL | 45 | 3.90 | 1.17 | 12 | 4.31 | 1.27 |
|  | SHARED | 45 | 4.19 | 1.26 | 5 | 3.93 | 1.92 |
|  | OPEN PLAN | 123 | 4.36 | 0.98 | 77 | 4.18 | 1.28 |
|  | FLEX/ABW | 59 | 4.09 | 0.97 | 13 | 4.46 | 1.14 |
| **Relational justice** | CELL | 46 | 2.78 | 1.04 | 12 | 2.90 | 1.12 |
|  | SHARED | 45 | 2.64 | 1.14 | 5 | 2.10 | 0.25 |
|  | OPEN PLAN | 124 | 2.89 | 0.86 | 76 | 2.83 | 0.84 |
|  | FLEX/ABW | 57 | 3.02 | 0.74 | 13 | 2.96 | 0.68 |
| **Trust in management** | CELL | 45 | 3.64 | 1.15 | 12 | 3.83 | 1.03 |
|  | SHARED | 45 | 3.49 | 1.10 | 5 | 2.80 | 1.10 |
|  | OPEN PLAN | 124 | 3.64 | 0.97 | 76 | 3.49 | 1.00 |
|  | FLEX/ABW | 59 | 3.95 | 0.78 | 13 | 3.92 | 0.76 |
| ^A^ Significant differences between the office types was seen in organisation A with both oneway ANOVA and Kruskal-Wallis.  ^B^ Significant differences between the office types was seen in organisation B with both oneway ANOVA and Kruskal-Wallis. | | | | | | | |
